# Glutathione‐Responsive Nanoparticles of Camptothecin Prodrug for Cancer Therapy

**DOI:** 10.1002/advs.202205246

**Published:** 2022-11-28

**Authors:** Lingpu Zhang, Lin Zhu, Lin Tang, Jiayi Xie, Yajuan Gao, Changyuan Yu, Kun Shang, Hongbin Han, Chaoyong Liu, Yunfeng Lu

**Affiliations:** ^1^ Beijing Advanced Innovation Center for Soft Matter Science and Engineering College of Life Science and Technology Beijing University of Chemical Technology Beijing 100029 P. R. China; ^2^ Department of Automatic Tsinghua University Peking University Third Hospital Beijing Key Laboratory of Magnetic Resonance Imaging Devices and Technology Beijing 100191 P. R. China; ^3^ Department of Radiology Peking University Third Hospital Institute of Medical Technology Peking University Health Science Center Beijing 100019 P. R. China

**Keywords:** cancer immunotherapy, controlled release, CPT prodrug, glutathione responsive, self‐assembly

## Abstract

Camptothecin (CPT) is a potent chemotherapeutic agent for various cancers, but the broader application of CPT is still hindered by its poor bioavailability and systemic toxicity. Here, a prodrug that releases CPT in response to glutathione (GSH), which is commonly overexpressed by cancer cells is reported. Through assembling with PEGylated lipids, the prodrug is incorporated within as‐assembled nanoparticles, affording CPT with a prolonged half‐life in blood circulation, enhanced tumor targetingability, and improved therapeutic efficacy. Furthermore, such prodrug nanoparticles can also promote dendritic cell maturation and tumor infiltration of CD8^+^ T cells, providing a novel strategy to improve the therapeutic efficacy of CPT.

## Graphical abstract

A camptothecin (CPT) prodrug is synthesized to assemble with PEGylated lipids as prodrug‐loaded nanoparticles, which can release CPT in response to overexpressed glutathione in cancer cells. Such prodrug nanoparticles endow CPT with a prolonged half‐life in the blood circulation and an enhanced immune response against cancer cells, resulting in significantly improved therapeutic efficacy.

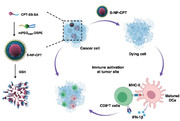

## 1 Introduction

Camptothecin (CPT),^[^ ^1^ ^]^ a DNA topoisomerase I inhibitor that induces DNA damage in cancer cells,^[^ ^2^ ^]^ has been widely used for the treatment of a variety of cancers,^[^ ^3^ ^]^ including colorectal and ovarian cancers.^[^ ^4^ ^]^ The efficacy of CPT, however, is still limited by its poor solubility, rapid hydrolysis, fast clearance, and systemic toxicity.^[^ ^5^ ^]^ To circumvent these limitations, various formulation technologies have been explored focusing mainly on the encapsulation of CPT within microspheres,^[^ ^6^ ^]^ microemulsions,^[^ ^7^ ^]^ polymer micelles,^[^ ^8^ ^]^ or polymer implants.^[^ ^9^ ^]^ Limited by the low solubility in pharmaceutically acceptable solvents, however, such formulations still have low loading of CPT.^[^ ^10^ ^]^ In addition, limited by the stability of such encapsulating architectures, premature release of CPT can occur.^[^ ^11^ ^]^ Developing approaches that enable effective delivery of CPT with reduced toxicity is of great interest to uncage the therapeutic power of CPT.

CPT has a planar pentacyclic ring structure containing an *α*‐hydroxylactone ring that plays an important role in both efficient topoisomerase I inhibition and in vivo potency.^[^ ^12^ ^]^ The lactone ring is highly susceptible to hydrolysis that leads to the formation of an inactive carboxylate.^[^ ^13^ ^]^ Furthermore, the *α*‐hydroxyl group induces an equilibrium that favors the inactive carboxylate form over the active lactone form under physiological conditions.^[^ ^5^, ^11^, ^14^ ^]^ Consequently, extensive efforts have been made to improve the hydrolysis stability and intracellular accumulation of CPT through increasing the lipophilicity of CPT, as well as modifying the *α*‐hydroxyl group.^[^ ^5^, ^15^ ^]^ Different groups (alkyl esters, amide, carbonate, etc.) were conjugated at the *α*‐hydroxyl site to stabilize the *α*‐hydroxylactone, improve the water solubility, and enhance the tumor targeting ability.^[^ ^3^ ^]^ Such derivatives (e.g., CRLX‐101 and EZ‐246) have entered clinical trials,^[^ ^16^ ^]^ although their potency is still limited by undesired pharmacokinetics and the ability to release authentic CPT.^[^ ^17^ ^]^

**Scheme 1:**
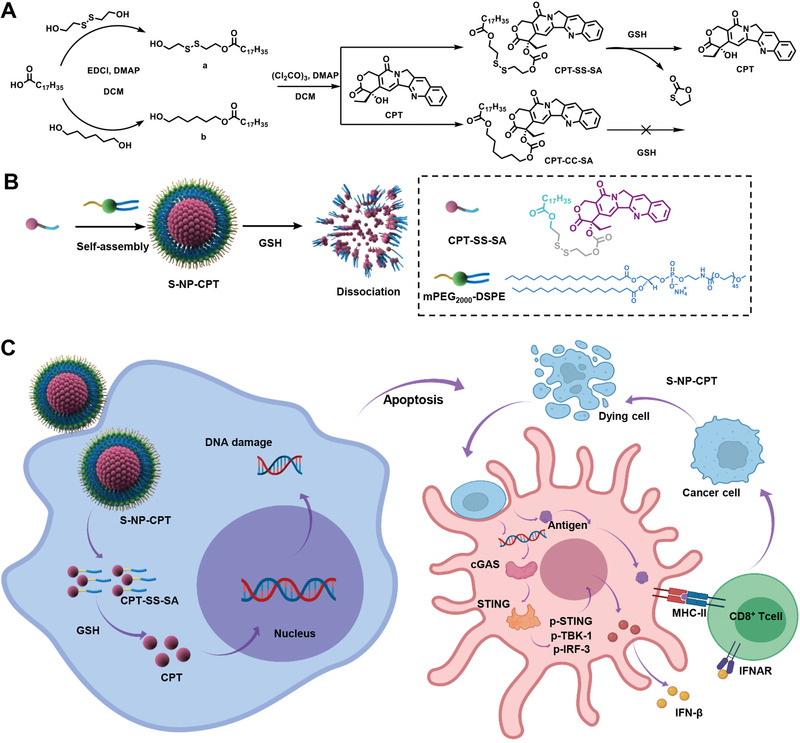
A) Synthetic routes of CPT‐SS‐SA. B) CPT‐SS‐SA (with disulfide bonds) could co‐assemble with PEG_2000_‐DSPE into S‐NP‐CPT (with GSH responsiveness). C) The possible mechanism of inducing immunogenic response upon treatment of S‐NP‐CPT in vitro.

We envision here that a prodrug, which can effectively release CPT in response to glutathione (GSH), can be synthesized through converting the *α*‐hydroxyl group to a disulfide‐containing stearic easter. As illustrated in **Scheme** **1**A, stearic acid (SA) was first reacted with 2‐hydroxyethyl disulfide to form SS‐SA (Figure S1, Supporting Information). The subsequent reaction of SS‐SA with CPT gives a CPT prodrug (CPT‐SS‐SA) (Figures S3 and S4, Supporting Information). In the presence of GSH, whose concentration in tumor cells is generally 100 to 1000 times higher than that in normal cells,^[^ ^18^ ^]^ the disulfide bond of CPT‐SS‐SA was cleaved and a sulfhydryl group was obtained. Subsequent nucleophilic reaction between the sulfhydryl group and the carbonyl group within the carbamate group resulted in the formation of a five‐member heterocyclic compound and the release of CPT.^[^ ^19^ ^]^ Furthermore, CPT‐SS‐SA contains a hydrophobic ester chain, which allows it to co‐assemble with lipids (e.g., distearoyl‐*sn*‐glycero‐3‐phosphoethanolamine‐*N*‐[methoxy(polyethylene glycol)‐2000] (mPEG_2000_‐DSPE) to form prodrug nanoparticles (S‐NP‐CPT, Scheme 1B). Similarly, CPT‐CC‐SA could self‐assemble with the lipids to form nanoparticles (C‐NP‐CPT). The PEG (polyethylene glycol) moieties in such PEGylated lipids could afford the CPT‐SS‐SA nanoparticles with prolonged blood circulation half‐life and the ability to effectively release CPT within tumors.

## 2 Results and Discussion

### 2.1 Synthesis of CPT‐SS‐SA and Characterization of S‐NP‐CPT

To obtain the prodrug, compound a (SA‐SS) containing disulfide bonds was synthesized by condensation of 2‐hydroxyethyl disulfide and SA (Scheme 1A). All of these compounds in the process were characterized by ^1^H NMR and ^13^C NMR (Figure S1, Supporting Information). For comparison, compound b (SA‐CC) without disulfide bonds was also designed (Scheme 1A) and characterized by ^1^H NMR and ^13^C NMR (Figure S2, Supporting Information). Subsequently, CPT‐SS‐SA and CPT‐CC‐SA were synthesized by further condensation of 2‐hydroxyethyl disulfide and SA (Scheme 1A), which were characterized by ^1^H NMR, ^13^C NMR, electrospray ionization mass spectrometry, and high‐performance liquid chromatography (HPLC; Figures S4–S7, Supporting Information).

**Figure 1:**
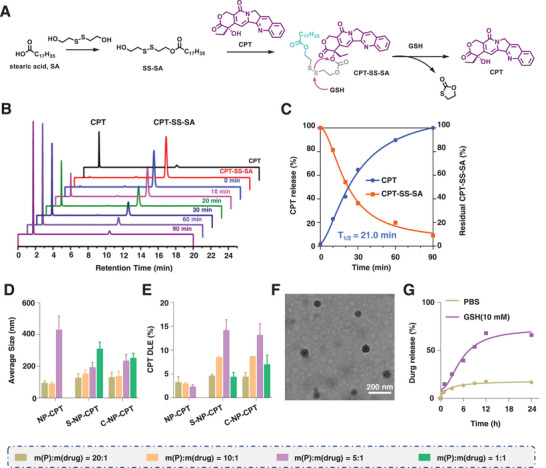
Characterization of CPT‐SS‐SA prodrug and S‐NP‐CPT. A) The mechanism of CPT release triggered by GSH. B,C) The CPT release profiles and kinetics of the CPT‐SS‐SA prodrug in the presence of GSH. D,E) The average sizes and drug loading efficiency (DLE) of CPT in S‐NP‐CPT at different feed ratios of mPEG_2000_‐DSPE to CPT‐SS‐SA. F) Representative TEM images of S‐NP‐CPT. G) The CPT release profiles of S‐NP‐CPT in PBS and in the presence of GSH (10 × 10^−3^ m).

Since CPT was released after breakdown of disulfide bonds in the presence of GSH, the release of CPT from the prodrug was first examined by HPLC by incubating CPT‐SS‐SA with GSH (5 × 10^−3^ m) at 37 °C. The results showed that the CPT‐SS‐SA peak at 10.47 min was found to decrease with increasing incubation time and disappeared after 90 min. Consistently, the CPT peak at 1.73 min appeared after 10 min of incubation and gradually increased with increasing incubation time, indicating a time‐dependent conversion of the prodrug to CPT in the presence of GSH (**Figure** **1**B). Further calculation of the conversion curve indicates that the half‐life of CPT‐SS‐SA in the presence of GSH was ≈21.0 min (Figure 1C).

Due to the presence of hydrophilic and hydrophobic components in mPEG_2000_‐DSPE, CPT‐SS‐SA with lipid chains provides hydrophobicity to form nanoparticles (S‐NP‐CPT) with mPEG_2000_‐DSPE (Scheme 1B). S‐NP‐CPT with different drug loadings was prepared in various feed mass ratios of CPT‐SS‐SA and mPEG_2000_‐DSPE at 1:20, 1:10, and 1:5, resulting in nanoparticles with an average diameter of 100–200 nm (Figure 1D), and a CPT loading capacity of 4.2%, 8.5%, and 13%, respectively (Figure 1E). For example, S‐NP‐CPT prepared with a mass feed ratio of CPT‐SS‐SA to mPEG_2000_‐DSPE at 1:10 had an average diameter of around 90 nm, which is consistent with the transmission electron microscopy (TEM) result (≈70 nm, Figure 1F). The polydispersity index for S‐NP‐CPT was about 0.15, and the zeta potential was around −17 mV (Figure S8B, Supporting Information). The stability of S‐NP‐CPT was evaluated by incubating S‐NP‐CPT in phosphate‐buffered saline (PBS) for 1 week. The results showed that the particle sizes remained almost unchanged during 7 days of incubation (Figure S8D, Supporting Information).

Due to its smaller size, S‐NP‐CPT prepared with a mass feed ratio of CPT‐SS‐SA to mPEG_2000_‐DSPE at 1:10 was used in the subsequent study. First, the dissociation of S‐NP‐CPT in the presence of GSH (10 × 10^−3^ m) was demonstrated by dynamic light scattering. The results demonstrated that the average size of S‐NP‐CPT shifted from 58.8 to 220 nm after GSH treatment (Figure S9, Supporting Information). Second, the CPT release from S‐NP‐CPT was further quantified by HPLC. The results showed that ≈60% and 17% CPT were released from S‐NP‐CPT within 24 h in the presence and absence of GSH, respectively (Figure 1G). These results suggested that CPT can be released rapidly from S‐NP‐CPT in response to GSH treatment.

### 2.2 In Vitro Cell Uptake and Antitumor Effect of S‐NP‐CPT

**Figure 2:**
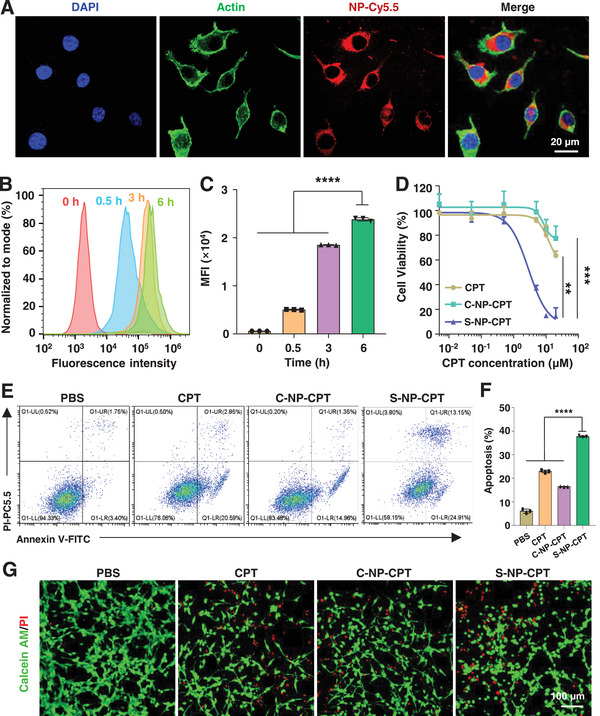
In vitro cell uptake and anticancer activity of S‐NP‐CPT. A) Representative CLSM images of CT26 cells after incubation with NP‐Cy5.5 for 3 h. The cell nucleus was stained with 4′,6‐diamidino‐2‐phenylindole (DAPI, blue). B,C) Flow cytometry was used to quantitatively evaluate the intracellular uptake of S‐NP‐CPT by CT26 cells. ****p < 0.0001 (ordinary one‐way analysis of variance (ANOVA)). D) Cell viability curves of various cell lines incubated with CPT and S‐NP‐CPT at 48 h. **p < 0.01, ***p < 0.001 (ordinary two‐way ANOVA). E,F) Cell apoptosis rates of CT26 cells with various treatments by flow cytometry. ****p < 0.0001 (ordinary one‐way ANOVA). G) Representative CLSM images of live (calcein acetoxymethyl ester, green signal)/dead (PI, red signal) assays of CT26 cells with various drug treatment. The green and red signals represented live and dead cells, respectively. Data represent mean ± standard deviation (SD) from *n* independent experiments (*n* = 3).

Intracellular uptake of S‐NP‐CPT by cancer cells was examined using a murine colorectal carcinoma cell line, namely, CT26 cells. S‐NP‐CPT was first labeled with a fluorescent dye Cy5.5 (defined as NP‐Cy5.5). To visualize the uptake of S‐NP‐CPT, confocal laser microscopy (CLSM) was used to observe the red fluorescence in CT26 cells that were treated with NP‐Cy5.5 for 3 h. The results showed that the red fluorescence in CT26 cells was mainly distributed in the cytoplasm (**Figure** **2**A), indicating that S‐NP‐CPT effectively entered CT26 cells. Further quantitative analysis by flow cytometry demonstrated that the fluorescence intensity in CT26 cells increased over time from 0.5 to 6 h, reaching a fourfold increase (Figure 2B,C). The intensity of the red fluorescence signal was also observed in cells treated with NP‐Cy5.5 with an increase in the incubation time. Together, these results collectively demonstrated that S‐NP‐CPT could effectively enter tumor cells in a time‐dependent manner.

To further investigate the cytotoxicity of S‐NP‐CPT, a 3‐(4, 5‐dimethylthiazolyl‐2)‐2, 5‐diphenyltetrazolium bromide assay was performed on CT26 cells. The cell viability curves for CPT and S‐NP‐CPT are shown in Figure 2D. The results showed that S‐NP‐CPT exhibited a more potent anticancer activity than native CPT in CT26 cells. Taking the CT26 cell line as an example, the IC_50_ value of S‐NP‐CPT in CT26 cells was 2.86 ± 0.12 × 10^−6^ m, which was significantly lower than that of CPT and S‐NP‐CPT (over 40 × 10^−6^ m). A further study of cell apoptosis revealed that S‐NP‐CPT induced the highest level of apoptosis in CT26 cells (Figure 2E), reaching 37.69 ± 0.33%; in contrast, at the same concentration of CPT and C‐NP‐CPT, the apoptosis rates were only 22.92 ± 0.54% and 16.44 ± 0.14%, respectively, indicating a significantly improved anticancer potency (Figure 2F). Subsequently, a calcein/propidium iodide (PI) live/dead assay kit was utilized to detect the cell killing effect. The calcein acetoxymethyl ester could stain live cells which emit green fluorescence, while PI stains dead cells that emit red fluorescence.^[^ ^20^ ^]^ CLSM results clearly showed that the untreated CT26 cells were mostly green, indicating that the cells were mostly alive. Moreover, cells treated with C‐NP‐CPT only showed little red fluorescence, indicating that a small number of cells died. However, CT26 cells treated with CPT and S‐NP‐CPT showed great red fluorescence, indicating that S‐NP‐CPT could cause strong toxicity in CT26 cells (Figure 2G).

### 2.3 In Vitro S‐NP‐CPT Could Activate the STING Pathway and Enhance the Antigen Presentation of DCs

**Figure 3:**
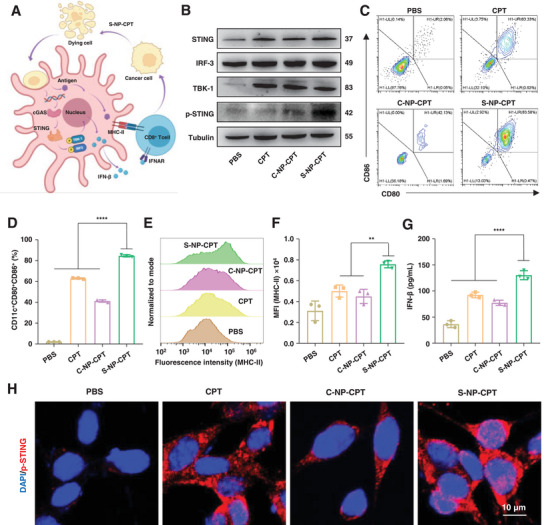
Activation of immune responses by S‐NP‐CPT in CT26 murine colon cancer cells by stimulating activation of the STING pathway in DCs. A) Schematic illustration of S‐NP‐CPT to activate the STING pathway in DCs. B) Proteins extracted from DC 2.4 cells after incubation with CT26 cells treated with PBS, CPT, C‐NP‐CPT, and S‐NP‐CPT for 24 h tested by WB. C,D) Semiquantitative study of activated DC 2.4 cells after incubation with CT26 cells treated with PBS, CPT, C‐NP‐CPT, and S‐NP‐CPT for 24 h by flow cytometry. ****p < 0.0001 (ordinary one‐way ANOVA). E,F) Semiquantitative study of MHC‐II expression in DC 2.4 cells by flow cytometry. **p < 0.01 (ordinary one‐way ANOVA). G) IFN‐*β* concentration in DC 2.4 cells supernatants after incubation with CT26 cells treated with PBS, CPT, C‐NP‐CPT, and S‐NP‐CPT for 24 h. ****p < 0.0001 (ordinary one‐way ANOVA). H) CLSM images of p‐STING upon various treatments. Cell nuclei were stained with DAPI (blue), and p‐STING was stained with Alexa Fluor 555 (red). Data represent mean ± standard deviation (SD) from *n* independent experiments (*n* = 3).

Currently, the tumor immune microenvironment has shown a great influence on cancer therapy.^[^ ^21^ ^]^ Increasing effective immune responses is one of the most critical approaches to reprogram the tumor immunosuppressive microenvironment. Activation of cGAS‐STING selectively could stimulate antigen‐presenting cells to initiate and activate tumor antigen‐specific T cells to infiltrate tumors. CPT induces DNA damage in tumor cells,^[^ ^15^ ^]^ which activates the interferon gene cyclic GMP‐AMP synthase stimulator (cGAS‐STING) pathway and generates an antitumor immune response.^[^ ^22^ ^]^ To further verify the activation of dendritic cells (DCs), DC 2.4 cells were incubated with the supernatant of CT26 cells pretreated with PBS, CPT, C‐NP‐CPT, or S‐NP‐CPT for 24 h. The DC 2.4 cells and the supernatant were then collected for further analysis. The expression of proteins in the STING pathway in DC 2.4 cells after various treatments was measured by Western blotting (WB). As shown in **Figure** **3**B, the supernatant of CT26 cells treated with S‐NP‐CPT significantly stimulated DC 2.4 cells to increase the expression of STING, phosphorylated STING (p‐STING), IRF‐3, and TBK‐1, indicating that CT26 cells treated with S‐NP‐CPT could activate the STING pathway of DCs.^[^ ^21^ ^]^ Flow cytometry was further applied to detect surface markers of DCs.^[^ ^23^ ^]^ As shown in Figure 3A, after incubation CT26 cells with S‐NP‐CPT, the expression levels of the costimulatory molecules CD80, CD86, and MHC‐II on the surface of DC 2.4 cells increased significantly, indicating the activation of DC 2.4 cells. CT26 cells treated with S‐NP‐CPT activated ≈80% population of DC 2.4 cells, which was significantly higher than those treated with C‐NP‐CPT (≈40%) and CPT (≈60%) (Figure 3C,D). Furthermore, CT26 cells treated with S‐NP‐CPT increased MHC‐II expression in DC 2.4 cells (Figure 3E,F) and stimulated DC 2.4 cells to secrete IFN‐*β* (Figure 3G).^[^ ^23^ ^]^ Furthermore, the ability of S‐NP‐CPT to activate the cGAS‐STING pathway was investigated by immunofluorescence through CLSM. The results showed that weak red fluorescence corresponding to overexpression of the STING protein was observed in CT26 cells after treatment with CPT and C‐NP‐CPT. However, a strong fluorescence signal was found after treatment with S‐NP‐CPT (Figure 3H and Figure S10, Supporting Information). Together, these results indicated that cancer cells can be captured by DCs after treatment with S‐NP‐CPT, which can subsequently stimulate the STING pathway, activate DCs, and enhance the antigen presentation of DCs.

### 2.4 In Vivo Biodistribution, Pharmacokinetics, and Biosafety Study

**Figure 4:**
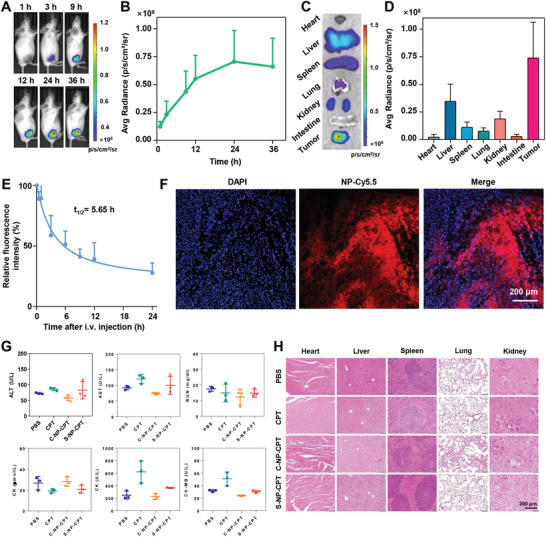
In vivo biodistribution, pharmacokinetics, and biosafety study of S‐NP‐CPT. A) In vivo fluorescence imaging (IVIS, Spectrum CT, PerkinElmer, *E*_x_/*E*_m_ = 650 nm/700 nm) of mice bearing CT26 cell tumors after iv injection of NP‐Cy5.5. B–D) Ex vivo images and semiquantitative study of excised organs and tumors at 36 h post‐injection. E) Blood circulation kinetics of NP‐Cy5.5 in mice with subcutaneous CT26 tumors after a single dose iv injection of NP‐Cy5.5. F) Representative CLSM images of tumor tissues in mice treated with NP‐Cy5.5. G) In vivo systemic toxicity evaluation after a single dose administration of drugs to healthy mice. ALT (alanine aminotransferase), AST (aspartate aminotransferase), BUN (blood urea nitrogen), CR (creatinine), CK (creatinine kinase), and CK‐MB (creatine kinase isoenzymes). H) In vivo systemic toxicity after single dose administration of drugs to healthy mice. H&E staining images of major organs dissected from treated mice.

The biodistribution and tumor accumulation of CPT were studied by intravenous injection of Cy5.5‐labeled NP‐Cy5.5. An increasing accumulation of nanoparticles was observed in the tumor after injection (**Figure** **4**A). The major organs and tumors were collected 36 h after injection. S‐NP‐CPT was found to accumulate mainly in the tumor, liver, and kidney, where the accumulation in the tumor is approximately twice that in the liver (Figure 4B–D). Meanwhile, CPT is a small molecule that is prone to being cleared in the blood circulation with a half‐life of ≈0.07 h in mice.^[^ ^22^ ^]^ In contrast, S‐NP‐CPT exhibited a prolonged half‐life of blood circulation of 5.65 h, indicating an 80‐fold improvement in blood circulation time (Figure 4E). Furthermore, the observation of the tumor tissue by CLSM indicated that the S‐NP‐CPT was evenly distributed within the tumor tissues, indicating deep tumor penetration of the nanoparticles (Figure 4F).

Furthermore, the systemic toxicity of S‐NP‐CPT was examined in healthy mice after one intravenous injection of various drugs (5 mg CPT kg^−1^). The results showed that the body weight of the mice remained unchanged for 14 days. Further physiological and biochemical indices of the blood, as well as hematoxylin and eosin (H&E) staining images of the main organs of mice treated with S‐NP‐CPT, were largely the same as those of mice treated with PBS, indicating a low toxicity of S‐NP‐CPT (Figure 4G,H).

### 2.5 In Vivo Antitumor Efficacy

**Figure 5:**
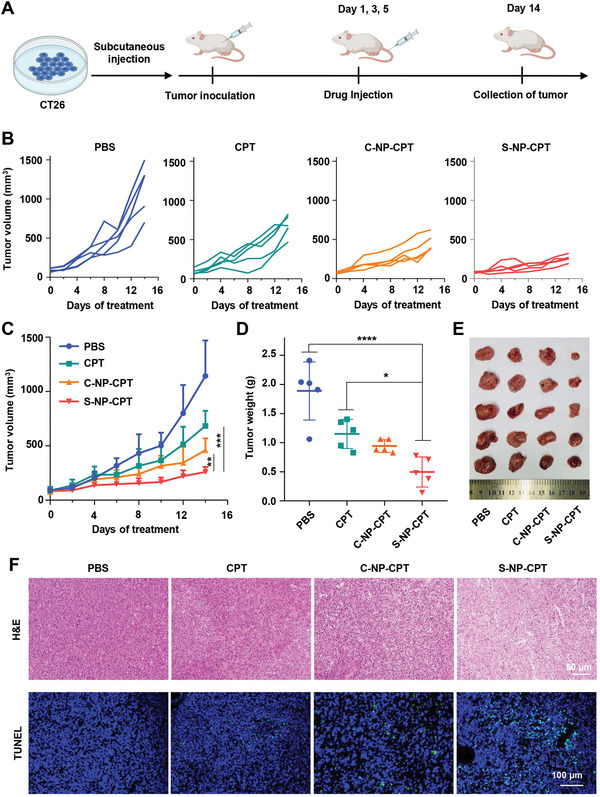
In vivo anticancer effect of S‐NP‐CPT on a CT26 murine colon cancer model. A) Schematic illustration of the establishment of the animal model and treatment schedules. B) The tumor volume of each group was monitored. C) Tumor growth inhibition curves of mice treated with PBS, CPT, C‐NP‐CPT, and S‐NP‐CPT, respectively. **p < 0.01, ***p < 0.001 (ordinary two‐way ANOVA). D,E) Ex vivo tumor weight and photographs of collected tumors after treatment on day 14. *p < 0.05, ****p < 0.0001 (ordinary one‐way ANOVA). F) H&E staining and TUNEL study of tumor tissues after treatment on day 14. Data represent mean ± standard deviation (SD) from *n* independent experiments (*n* = 5).

The antitumor effect of S‐NP‐CPT was then studied in mice with CT26 tumors. When the tumor size reached 100 mm^3^, 15 mice with CT26 tumors were randomly divided into three groups (*n* = 5), after which they were injected intravenously with PBS (control group), CPT, C‐NP‐CPT, and S‐NP‐CPT (5 mg of CPT per kg of body weight), respectively (**Figure** **5**A). The tumor volume of each group was monitored (Figure 5B). The results showed that mice treated with CPT and C‐NP‐CPT showed limited tumor growth, resulting in a tumor inhibition rate of 43% and 64%, respectively, while S‐NP‐CPT had a more significant tumor suppression effect, with an inhibition rate of 79% (Figure 5C). Specifically, on day 14 the tumors of each mouse were collected and weighed. The mean tumor weight in mice treated with S‐NP‐CPT was 0.5 ± 0.25 g, which was significantly less than that of mice treated with C‐NP‐CPT (0.94 ± 0.11 g) and CPT (1.15 ± 0.24 g) (Figure 5D). Therefore, S‐NP‐CPT had a more potent antitumor effect than CPT (Figure 5E). Furthermore, H&E staining and terminal deoxynucleotidyl transferase dUTP nick end labeling (TUNEL) assay were performed on the tumor slices. The results showed that the tumors in the mice treated with S‐NP‐CPT showed larger areas of apoptosis, indicating there was a higher apoptosis rate in the tumors (Figure 5F).

### 2.6 S‐NP‐CPT Enhances CD8^+^T Cell Infiltration and DC Maturation

**Figure 6:**
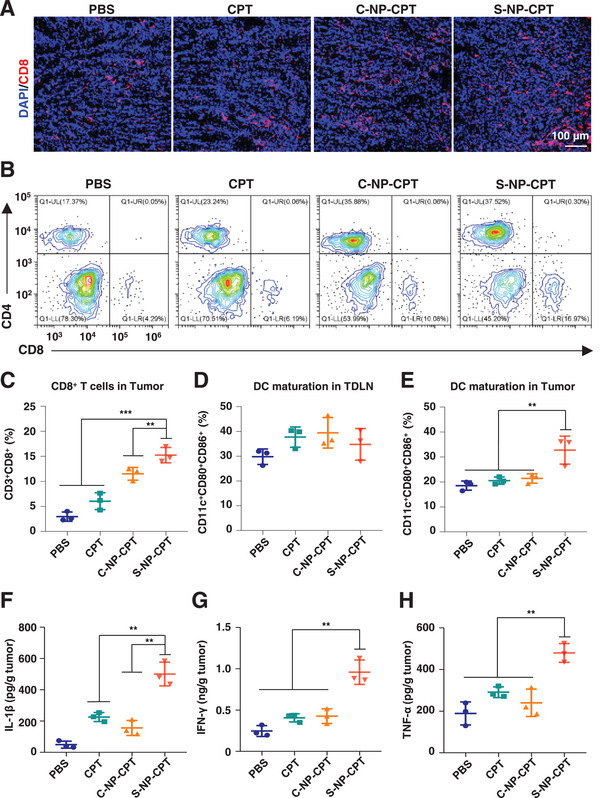
Activation of cancer immunity by S‐NP‐CPT in a CT26 murine colon cancer model. A) Immunofluorescence imaging of CD8^+^ T cells (red) in CT26 tumor tissues. The cell nuclei were stained with DAPI (blue). B,C) Representative flow cytometric plots of T cells in tumor tissues gating on CD3^+^ T cells in each group (*n*  =  3). D,E) Representative flow cytometric analysis of activated dendritic cells (CD11c^+^CD80^+^CD86^+^) in TDLN and tumor. F–H) Cytokine levels (IL‐1*β*, IFN‐*γ*, and TNF‐*α*) in tumor tissues were detected by ELISA kit. **p < 0.01, ***p < 0.001 (ordinary one‐way ANOVA). Data represent mean ± standard deviation (SD) from *n* independent experiments (*n* = 3).

Encouraged by the above impressive results, the immune response was subsequently investigated in a mouse model by analyzing tumor tissues from the treated mice. Tumor sections were further stained with a CD8 antibody for immunofluorescence imaging. Compared to CPT, S‐NP‐CPT resulted in an increased intensity of red fluorescence within the tumor sections, suggesting that there was an increased tumor infiltration of CD8^+^ T cells (**Figure** **6**A). Consistently, CD8^+^ T cells (CD3^+^CD8^+^) in tumor tissues of mice treated with S‐NP‐CPT were approximately five times higher than those of mice treated with PBS (15% vs 3%) (Figure 6B,C and Figure S8, Supporting Information). Furthermore, S‐NP‐CPT was more effective in promoting DC maturation (CD11c^+^CD80^+^CD86^+^), reaching a DC maturation rate of 32.75 ± 1.53% of the entire population of DC cells, which is much more effective than CPT (Figure 6D,E and Figure S11, Supporting Information). Such an increased infiltration of CD8^+^ T cells and DC maturation in the tumor could lead to altered levels of cytokines (IL‐1*β*, TNF‐*α*, and IFN‐*γ*), which are involved in the regulation of antitumor immune. As expected, additional cytokine‐related enzyme‐linked immunosorbent assay (ELISA) results showed that cytokine levels (IL‐1*β*, TNF‐*α*, and IFN‐*γ*) in tumor tissues increased after S‐NP‐CPT treatment (Figure 6F–H), indicating that mice treated with S‐NP‐CPT had an elevated antitumor immune response.

## 3 Conclusion

In conclusion, we have successfully developed a CPT prodrug that can self‐assemble with a PEGylated lipid to form prodrug nanoparticles and release CPT in response to GSH. This strategy significantly prolonged the half‐life of the blood circulation, increased tumor targeting capacity, and improved the therapeutic efficacy of CPT. Furthermore, such prodrug nanoparticles could cause DNA damage to activate the STING pathway and stimulate a powerful immune response, which could then induce the maturation of DCs and tumor infiltration of CD8^+^ T cells in tumors, resulting in the elicitation of antitumor immunity in vivo and finally transforming “immune cold tumors” into “immune hot tumors” for combined chemotherapy and cancer immunotherapy. This work provides a novel design for cancer therapy using CPT.

## Conflict of Interest

The authors declare no conflict of interest.

## Supporting information

Supporting Information

## Data Availability

The data that support the findings of this study are available from the corresponding author upon reasonable request.
