# Supplementary material for: Glutathione‐Responsive Nanoparticles of Camptothecin Prodrug for Cancer Therapy
Source: Adv Sci (Weinh). 2022 Nov 28;10(3):2205246. doi: 10.1002/advs.202205246 (PMC9875659; doi:10.1002/advs.202205246)
Supplement: Supplementary file 1 — Supporting Information [file ADVS-10-2205246-s001.pdf]

ADVANCED SCIENCE
Open Access

Supporting Information

for *Adv. Sci.*, DOI 10.1002/advs.202205246

Glutathione-Responsive Nanoparticles of Camptothecin Prodrug for Cancer Therapy

Lingpu Zhang, Lin Zhu, Lin Tang, Jiayi Xie, Yajuan Gao, Changyuan Yu, Kun Shang, Hongbin Han*, Chaoyong Liu* and Yunfeng Lu*

Supporting Information

Glutathione-Responsive Nanoparticles of Camptothecin Prodrug for Cancer Therapy

Lingpu Zhang, Lin Zhu, Lin Tang, Jiayi Xie, Yajuan Gao, Changyuan Yu, Kun Shang, Hongbin Han,* Chaoyong Liu,* Yunfeng Lu*

L Zhang, L Zhu, L Tang, Prof. C. Yu, Prof. C. Liu, Prof. Y. Lu

Beijing Advanced Innovation Center for Soft Matter Science and Engineering,

College of Life Science and Technology, Beijing University of Chemical Technology,

Beijing 100029, China

Email: chaoyongliu@mail.buct.edu.cn

J Xie

Department of Automatic, Tsinghua University, Peking University Third Hospital, Beijing Key Laboratory of Magnetic Resonance Imaging Devices and Technology, Beijing 100191, China

Y Gao, K. Shang, Prof. H. Han

Department of Radiology, Peking University Third Hospital, Institute of Medical Technology, Peking University Health Science Center, Beijing 100019, China

Email: shangkun@bjmu.edu.cn; hanhongbin@bjmu.edu.cn

Contents

Experimental section

Figure S1. ^{1}H NMR and ^{13}C NMR of compound a.

Figure S2. ^{1}H NMR and ^{13}C NMR of compound b.

Figure S3. ^{1}H NMR and ^{13}C NMR of CPT-SS-SA.

Figure S4. ESI-MS spectrum of CPT-SS-SA.

Figure S5. ^{1}H NMR and ^{13}C NMR of CPT-CC-SA.

Figure S6. ESI-MS spectrum of CPT-CC-SA.

Figure S7. Characterization of S-NP-CPT.

Figure S8. Representative particle size distribution of S-NP-CPT after GSH treatment.

Figure S9. Gating strategy for flow cytometry analysis.

References

Experimental section

Materials and Measurements

Materials: $mPEG_{2000}$-DSPE was purchased from Alfa Aesar. Camptothecin (CPT), 3-(4,5-dimethylthiazol-2-yl)-2,5-diphenyltetrazolium bromide (MTT), triphosgene, 4-dimethylaminopyridine, stearic acid (SA), sodium dodecyl sulfate (SDS) were purchased from Aladdin, Shanghai. 2-hydroxyethyl disulfide, 1,6-hexanediol were purchased from InnoChem, Beijing. 1-ethyl-3-(3-dimethyllaminopropyl) carbodiimide hydrochloride (EDC·HCl), chloroform-d ($CDCl_3$) were purchased from Energy-chemical, Shanghai. 2-(4-amidinophenyl)-1H-indole-6-carboxamidine (DAPI), Cy5.5, Cy7.5 and FITC Phalloidin were purchased from Solarbio, Beijing. The Annexin V-FITC/PI cell apoptosis kit and the Calcein/PI Live/Dead Viability/Cytotoxicity Assay Kit were purchased from Beyotime Biotechnology, Shanghai. RPMI-1640 medium, 0.25% trypsin-EDTA, fetal bovine serum (FBS) and penicillin/streptomycin (P/S) were purchased from Gibco (Gran Island, NY, USA). PE anti-mouse CD11c, FITC anti-mouse CD80, APC anti-mouse CD86, PerCP/Cyanine5.5 anti-mouse CD3, FITC anti-mouse CD8a, PerCP/Cyanine5.5 anti-mouse I-A/I-E Antibody, APC anti-mouse CD4 were purchased from BioLegend (USA).

Instrumentation and Methods

^{1}H- and ^{13}C-NMR spectra were recorded on a 400 MHz NMR spectrometer (Bruker). Chemical shifts (δ) are reported in parts per million (ppm) referenced to tetramethylsilane (δ 0.00) ppm using the solvent peaks of the residual protons as internal standards. Transmission electron microscopy images were recorded on an HT-7700 transmission electron microscope (Hitachi, Japan). Dynamic light scattering measurements were performed on a Malvern Zetasizer Nano ZS90 laser particle size analyzer (Nano ZS, UK). Flow cytometry analysis was performed on a CytoFLEX Flow Cytometer instrument (Becton Dickinson, San Jose, CA, USA).

Ethical Approval

All animal experiments were conducted according to the ethical regulations for animal testing and received approval from the Peking University Institutional Animal Care and Use Committee (LA2021316).

Cell lines and animals: The CT26 cells were cultured in RPMI-1640 medium. Balb/c mice (female, 4 weeks old) were purchased from Vital River Laboratory Animal Technology Co. Ltd. (Beijing, China) and raised in SPF animal rooms.

Synthesis of SA-SS (a): SA (2.84 g, 10 mmol), EDC·HCl (2.30 g, 12 mmol), 4-dimethylaminopyridine (122.17 mg, 1mmol) were dissolved in 30 mL of ultradry dichloromethane. After stirring for 10 min, 2-hydroxyethyl disulfide (1.54 g, 10 mmol) was dissolved in 10 mL of ultra-dry dichloromethane, added dropwise to the reaction mixture and stirred at room temperature overnight. The reaction solution was washed with hydrochloric acid, then dried with anhydrous sodium sulfate, and sodium sulfate was removed, and finally the sample was separated by column chromatography of silica gel to obtain SA-SS (a). SA-SS was characterized by nuclear magnetic resonance (NMR).

^{1}H NMR (400 MHz, Chloroform-d) δ 4.35 (t, *J* = 6.7 Hz, 2H), 3.89 (t, *J* = 5.8 Hz, 2H), 2.91 (dt, *J* = 15.5, 6.2 Hz, 4H), 2.32 (t, *J* = 7.5 Hz, 2H), 1.62 (p, *J* = 7.6 Hz, 2H), 1.25 (s, 28H), 0.88 (t, *J* = 6.7 Hz, 3H). ^{13}C NMR (101 MHz, $CDCl_3$) δ 173.73, 62.14, 60.24, 41.66, 37.10, 34.22, 31.92, 29.69, 29.65, 29.60, 29.46, 29.36, 29.25, 29.13, 24.91, 22.69, 14.11.

Synthesis of SA-CC (b): SA (2.84 g, 10 mmol), EDC·HCl (2.30 g, 12 mmol), 4-dimethylaminopyridine (122.17 mg, 1mmol) were dissolved in 30 mL of ultradry dichloromethane. After stirring for 10 min, 1,6-hexanediol (1.77 g, 15 mmol) was dissolved in 10 ml of ultra-dry dichloromethane, added dropwise to the reaction mixture and stirred at room temperature overnight. The reaction solution was washed with hydrochloric acid, then dried with anhydrous sodium sulfate, and sodium sulfate was removed, and finally the sample was separated by silica gel column chromatography to obtain SA-CC (b). SA-CC was characterized by nuclear magnetic

resonance (NMR).

^{1}H NMR (400 MHz, Chloroform-*d*) δ 4.35 (t, *J* = 6.7 Hz, 2H), 3.89 (t, *J* = 5.8 Hz, 2H), 2.91 (dt, *J* = 15.5, 6.2 Hz, 4H), 2.32 (t, *J* = 7.5 Hz, 2H), 1.62 (p, *J* = 7.6 Hz, 2H), 1.25 (s, 28H), 0.88 (t, *J* = 6.7 Hz, 3H). ^{13}C NMR (101 MHz, $CDCl_3$) δ 174.02, 77.36, 77.04, 76.72, 64.19, 62.75, 34.39, 32.60, 31.93, 29.70, 29.66, 29.61, 29.48, 29.37, 29.28, 29.17, 28.63, 25.74, 25.39, 25.01, 22.69, 14.11.

Synthesis of CPT-SS-SA: CPT (610.58 mg, 1.75 mmol), EDC·HCl (640.50 mg, 5.25 mmol) were dissolved in 10 mL of ultra-dry dichloromethane. Under nitrogen protection, triphosgene (173.60 mg, 0.58 mmol) was added to the reaction mixture in batches. After stirring for 30 minutes at room temperature, SA-SS (a) (814.00 mg, 1.93 mmol) was dissolved in a small amount of dichloromethane and dropped into the reaction mixture. After 16 h, the reaction mixture was washed with hydrochloric acid and dried with anhydrous sodium sulfate, then sodium sulfate was removed and finally CPT-SS-SA was separated by silica gel column chromatography. CPT-SS-SA was characterized by nuclear magnetic resonance (NMR).

^{1}H NMR (300 MHz, Chloroform-*d*) δ 8.41 (s, 1H), 7.95 (d, *J* = 8.1 Hz, 1H), 7.85 (t, *J* = 7.0 Hz, 1H), 7.34 (s, 1H), 5.71 (d, *J* = 17.3 Hz, 1H), 5.40 (d, *J* = 17.3 Hz, 1H), 5.30 (s, 2H), 4.37 (td, *J* = 6.8, 3.1 Hz, 2H), 4.26 (t, *J* = 6.5 Hz, 2H), 2.91 (dt, *J* = 16.8, 6.6 Hz, 4H), 2.22 (dt, *J* = 27.8, 7.2 Hz, 4H), 1.30-1.19 (m, 28H), 1.01 (t, *J* = 7.5 Hz, 3H), 0.88 (t, *J* = 6.5 Hz, 3H). ^{13}C NMR (75 MHz, $CDCl_3$) δ 173.51, 167.24, 157.24, 153.44, 152.25, 148.84, 146.49, 145.59, 131.17, 130.69, 129.64, 128.47, 128.19, 128.17, 128.07, 120.20, 95.94, 78.03, 77.53, 77.10, 76.68, 67.05, 66.54, 61.93, 50.01, 37.29, 36.53, 34.12, 31.91, 31.85, 29.69, 29.64, 29.59, 29.45, 29.35, 29.25, 29.11, 24.86, 22.68, 14.13, 7.65.

Synthesis of CPT-CC-SA: CPT (768 mg, 2 mmol), and EDC·HCl (732 mg, 6 mmol) were dissolved in 10 mL of ultra-dry dichloromethane. Under nitrogen protection, triphosgene (173.60 mg, 0.58 mmol) was added to the reaction in batches. After stirring for 30 min at room temperature, SA-CC (b) (844.80 mg, 2.2 mmol) was

dissolved in a small amount of dichloromethane and dropped into the reaction mixture. After 16 h, the reaction mixture was washed with hydrochloric acid and dried with anhydrous sodium sulfate, then sodium sulfate was removed and finally CPT-CC-SA was separated by silica gel column chromatography. CPT-CC-SA was characterized by nuclear magnetic resonance (NMR).

^{1}H NMR (400 MHz, Chloroform-*d*) δ 8.41 (s, 1H), 8.23 (d, *J* = 8.5 Hz, 1H), 7.95 (dd, *J* = 8.3, 1.3 Hz, 1H), 7.90-7.80 (m, 1H), 7.68 (ddd, *J* = 8.1, 6.8, 1.2 Hz, 1H), 7.34 (s, 1H), 5.70 (d, *J* = 17.3 Hz, 1H), 5.40 (d, *J* = 17.2 Hz, 1H), 5.35-5.24 (m, 2H), 4.12 (ddt, *J* = 28.3, 10.7, 6.6 Hz, 2H), 3.99 (t, *J* = 6.6 Hz, 2H), 2.35-2.09 (m, 4H), 1.76-1.50 (m, 8H), 1.25 (d, *J* = 2.1 Hz, 31H), 1.00 (t, *J* = 7.5 Hz, 3H), 0.88 (t, *J* = 6.8 Hz, 3H). ^{13}C NMR (75 MHz, $CDCl_3$) δ 173.91, 167.45, 157.26, 153.79, 152.29, 148.81, 146.38, 145.79, 131.20, 130.69, 129.56, 128.46, 128.21, 128.15, 128.05, 120.25, 95.98, 77.54, 77.11, 76.69, 68.96, 67.03, 64.06, 49.97, 34.31, 31.90, 29.67, 29.63, 29.59, 29.46, 29.34, 29.25, 29.14, 28.44, 28.41, 25.54, 25.26, 24.97, 22.67, 14.12, 7.64.

Determination of the reaction kinetics of CPT-SS-SA with GSH: An aqueous solution of SA-SS-CPT (10 μM) was combined with glutathione (GSH, 5 mM) (Acetonitrile: DMSO = 9: 1). At specified time points, 10 μL of the reaction mixture were extracted and injected into an HPLC column (Agilent 1260, Agilent Technologies, USA Diamonsi C18 column (250 mm x 4.6 mm, 5 μm), acetonitrile as mobile phase, flow rate 1 mL/min, detection wavelength 375 nm, column temperature 30 °C).[1]

Preparation and characterization of S-NP-CPT: $mPEG_{2000}$-DSPE (10 mg) and CPT-SS-SA (1 mg) were dissolved in 5 ml of mL methyl trichloride, and the chloroform solution was placed in a round bottom flask and rotatably evaporated, forming a film on the inner wall of the flask. Then 10 mL of distilled water was added and sonicated for 10 min. Subsequently, the supernatant S-NP-CPT was collected by centrifugation (5,000 rpm. 5 min). C-NP-CPT was prepared in a manner similar to that described above. The CPT content in S-NP-CPT was determined by HPLC.

The morphology of S-NP-CPT was characterized by TEM (HT-7700, Hitachi, Japan). The size of S-NP-CPT was characterized by the Malvern Zetasizer Nano ZS90 laser particle size analyzer (Nano ZS, UK).

In *vitro* cellular uptake of S-NP-CPT by CLSM and flow cytometry: mPEG$_{2000}$-DSPE (10 mg), CPT-SS-SA (1 mg) and Cy5.5 (1 mg) were dissolved in 5 mL of methyl trichloride, and then the chloroform solution was placed in a round bottom flask on a rotatable evaporator, forming a film on the inner wall of the flask. Then 10 mL of distilled water was added, which was further sonicated for 10 min. The supernatant (NP-Cy5.5) was collected by centrifugation (5,000 rpm. 5 min).

Intracellular uptake of NP-Cy5.5 was studied by CLSM and flow cytometry. For CLSM observation, CT26 cells were seeded in 24-well chamber slides (Thermo Scientific, USA) at a density of 2×10^4 cells per well and incubated with RPMI1640 supplemented with 10% FBS (1 mL) at 37 °C for 12 h. After removing the medium, cells were treated with NP-Cy5.5 at an equivalent Cy5.5 concentration (10 μg/mL) for 0.5 h, 3 h, and 6 h, respectively. The medium was removed and cells were incubated with FITC phalloidin according to the manufacturer’s protocol. Subsequently, the cells were stained with DAPI and then observed by laser confocal microscope (OLYMPUS FV1000-IX81, Olympus, Japan). For flow cytometry, CT26 cells were seeded in 12-well plates (2×10^5 cells/well) and cultured for 12 h. After treatment with NP-Cy5.5 at an equivalent Cy5.5 concentration (10 μg/mL) for 0.5, 3 and 6 h, cells were collected and tested by flow cytometry (Becton Dickinson and Company, USA).

Cell variability of S-NP-CPT in cancer cells: The MTT assay was used to examine the cytotoxicity of S-NP-CPT in CT26 cells. The cells were seeded in 96-well plates (Thermo Scientific, USA) at a density of 8000 cells per well and cultured for 12 h. Cells were incubated with CPT, S-NP-CPT, C-NP-CPT with a concentration of CPT ranging from 0.05, 0.5, 5, 10, 20 to 40 μM for 48 h, respectively. Then 10% MTT diluted with RPMI1640 (100 μL) was added to the wells. After incubation at 37 °C, 10% SDS (100 μL) was added to each well and incubated at 37 °C for another 12 h.

The absorbance of the well was tested by a Microplate reader (SpectraMax) at 570 nm (peak absorbance) and subtracted at 650 nm (background absorbance). Cell viability was expressed as the ratio of the absorbance of the test and control wells.

Cell apoptosis of S-NP-CPT in CT26 cells: CT26 cells were seeded in 6-well plates (Thermo Scientific, USA) at a density of 2×10^5 cells per well and cultured for 12 h. Cells were incubated with CPT, S-NP-CPT, C-NP-CPT at a final concentration of CPT at 10 μM for 24 h. Cells were collected and stained with Annexin V-FITC/PI cell apoptosis kit following the manufacturer's instructions. The samples were finally analyzed via flow cytometry (Becton Dickinson and Company, USA).

***In Vivo* FL Imaging and Biodistribution Analysis:** CT26 cells (1×10^6) were subcutaneously injected into the right hip of female BALB/c mice. When tumor volumes reached about 100 mm^3, mice were injected with NP-Cy5.5 iv. After injection, FL signals were recorded using the IVIS spectrum imaging system (Spectrum CT, PerkinElmer) at 1, 3, 9, 12, 24, 36 h, respectively. For the biodistribution study, mice were sacrificed after 36 h after injection and tumors and normal organs were harvested and imaged.

Pharmacokinetic study of S-NP-CPT: CT26 cells (1×10^6) were subcutaneously injected into the right hip of female BALB/c mice. When tumor volumes reached about 100 mm^3, mice were injected with NP-Cy5.5 *iv.* After injection, at each required time point (5 min, 0.5, 1, 3, 6, 9, 12, 24 h) 10 μL of plasma was collected from the tail vein and then the concentration of Cy5.5 in the plasma was measured by fluorescence. Subsequently, the tumors were sectioned and observed by CLSM.

Activation of immune responses by S-NP-CPT *through stimulation of activation of the* STING pathway in DCs: DC2.4 cells were incubated with CT26 cells treated with PBS, CPT, C-NP-CPT, and S-NP-CPT for 24 h. DC2.4 cells and supernatant were collected for subsequent analysis.

Flow cytometry analysis: The DC2.4 cells above were resuspended and blocked with 0.1% BSA in PBS, and stained with the corresponding antibody (PE anti-mouse

CD11c, FITC anti-mouse CD80, APC anti-mouse CD86, PerCP/Cyanine5.5 anti-mouse I-A/I-E antibody, Biolegend, USA) prepared in 0.1% BSA in PBS for 1 h at room temperature. Finally, cells were detected by flow cytometry (Beckman Coulter, U.S.A).

Establishment of CT26 solid tumor model and evaluation of therapeutic effect: CT26 cells (1×10^6) were subcutaneously injected into the right flank of female BALB/c mice. The mice were injected by iv with PBS, CPT, C-NP-CPT, and S-NP-CPT at a dose of 5 mg CPT kg^{-1}, respectively, when tumor size reached 100 mm^3. Tumor volume was recorded every other day.

Tumor immune microenvironment analysis: Mice exhibiting CT26 tumor were randomly divided into six groups (3 mice in each group) when the tumor size reached approximately 100 mm^3. The groups received different treatments, for example, PBS, CPT, C-NP-CPT and S-NP-CPT at an ac dose of 5 mg of CPT kg^{-1}. After 3 days of treatment, the tumor tissues and lymphatics were digested. The samples were then resuspended and blocked with 0.1% BSA in PBS and stained with the corresponding antibody prepared in 0.1% BSA in PBS for 1 h at room temperature. Finally, it was detected by flow cytometry (FCM, Beckman Coulter, U.S.A).[2]

Statistical analysis：The level of significance in all statistical analyses was set at $P < 0.05$. Data were analyzed using a two-sided Student’s t-test when two groups were being compared. One-way or two-way analysis of variance (ANOVA) and Tukey posthoc tests were used when more than two groups were compared (multiple comparisons) using Prism 8.0 (GraphPad Software).

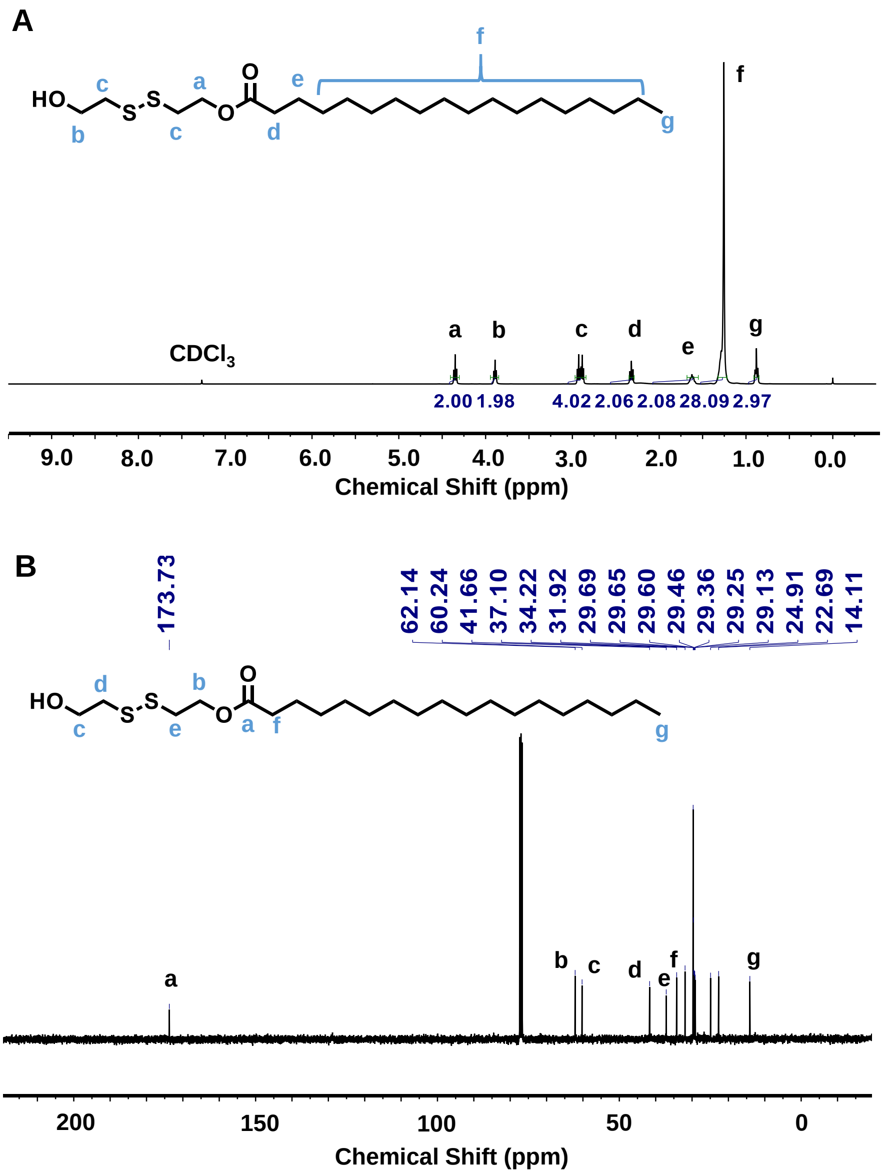

Figure S1. ^{1}H NMR and ^{13}C NMR of compound a.

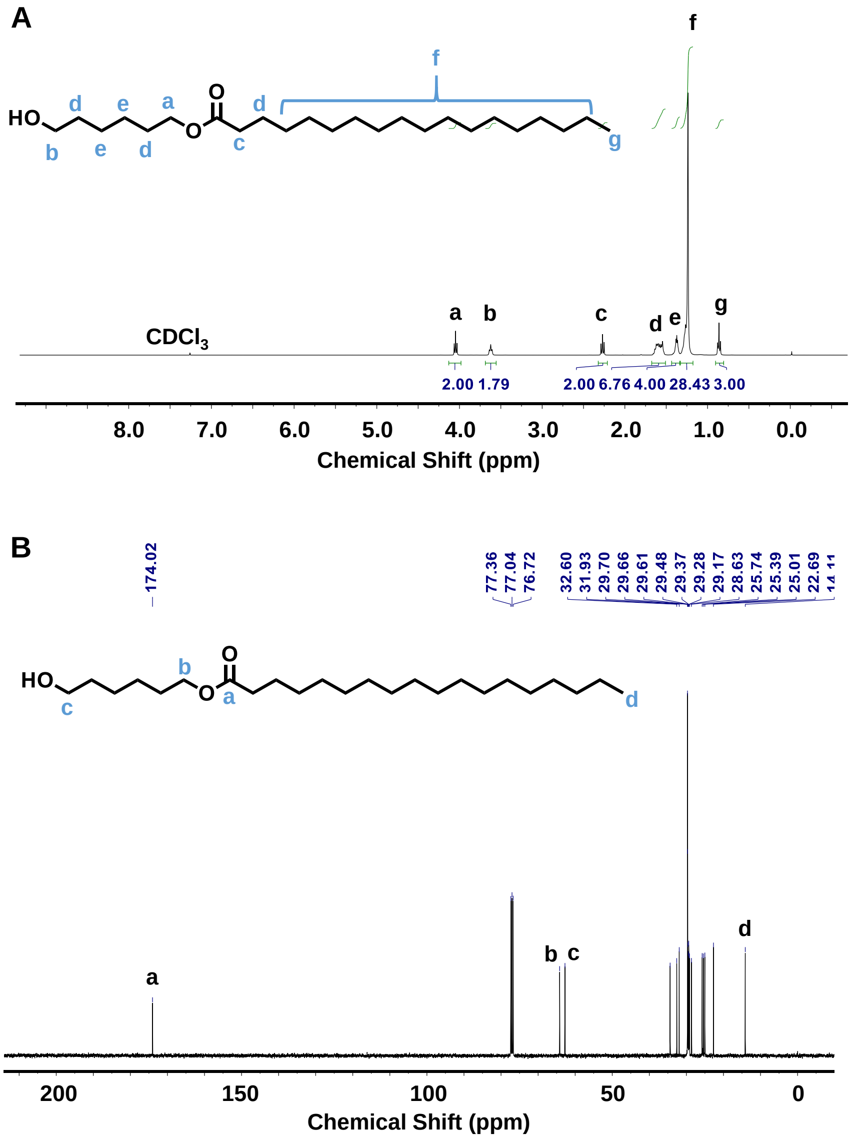

Figure S2. ^{1}H NMR and ^{13}C NMR of compound b.

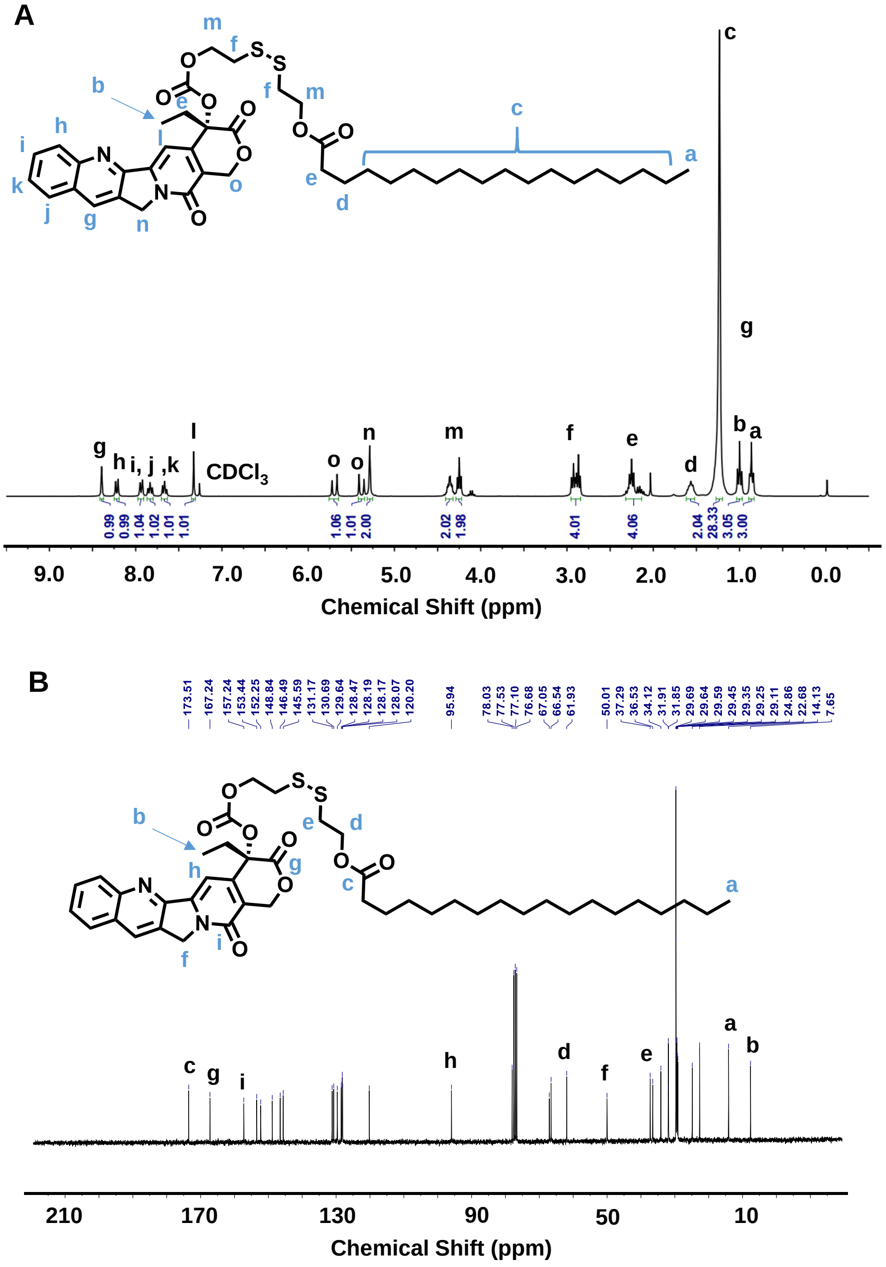

Figure S3. ^{1}H NMR and ^{13}C NMR of CPT-SS-SA.

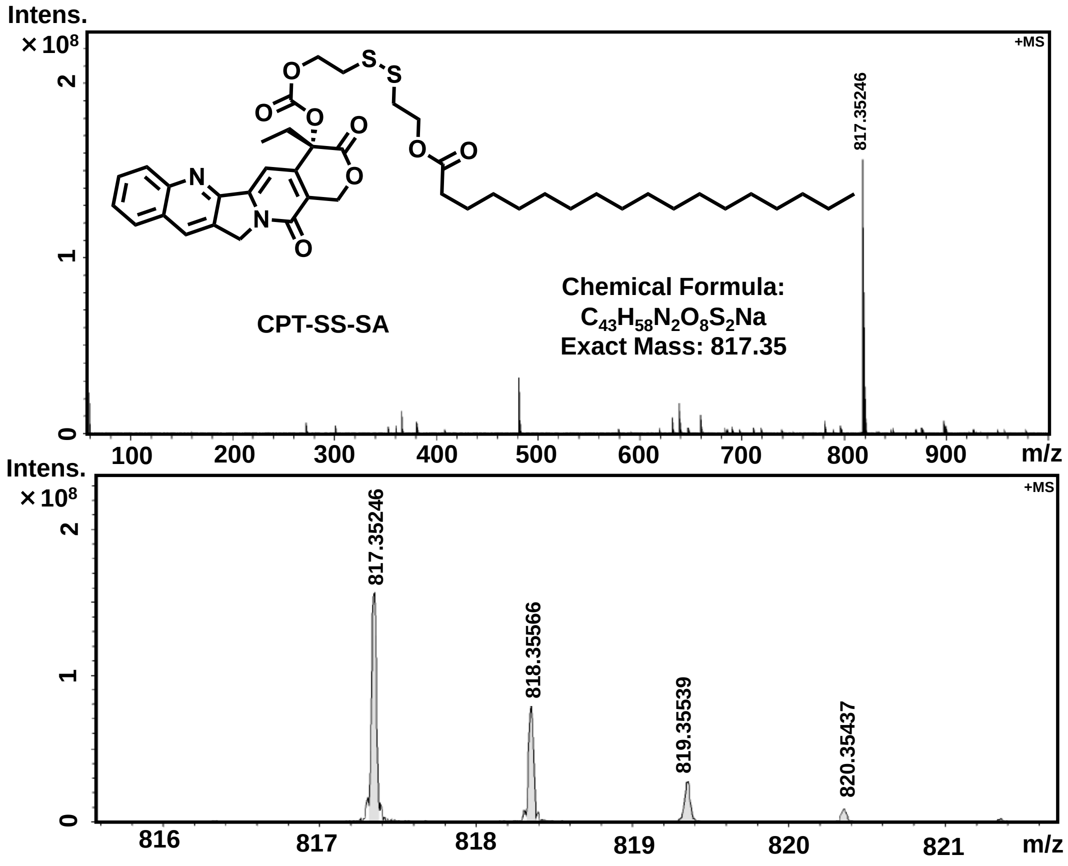

Figure S4. ESI-MS spectrum of CPT-SS-SA.

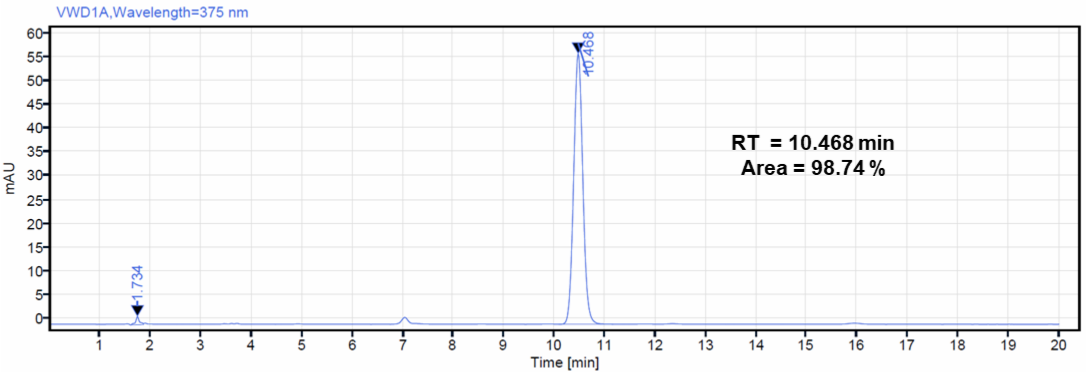

Figure S5. HPLC chromatogram of CPT-SS-SA.

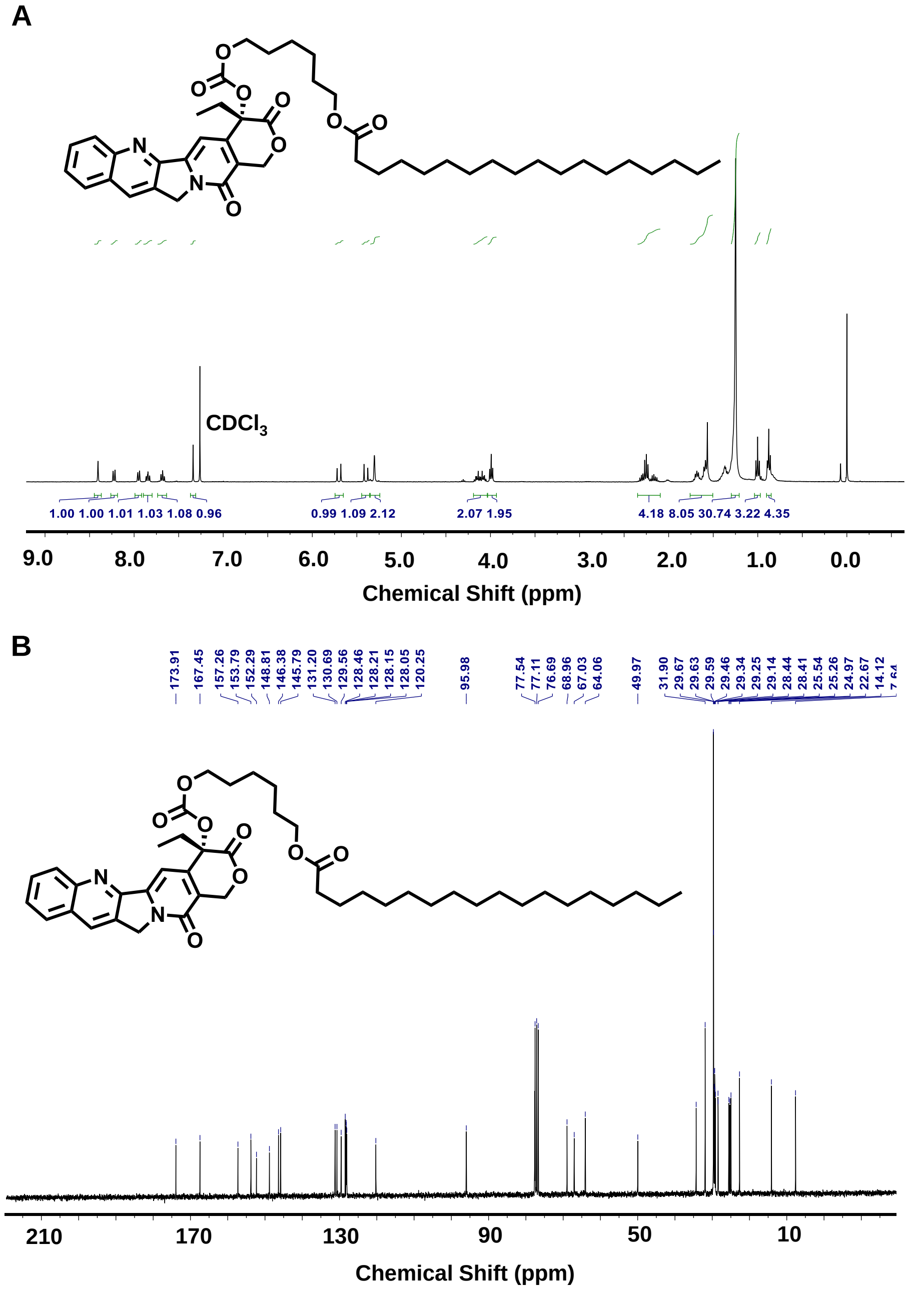

Figure S6. ^{1}H NMR and ^{13}C NMR of CPT-SS-SA.

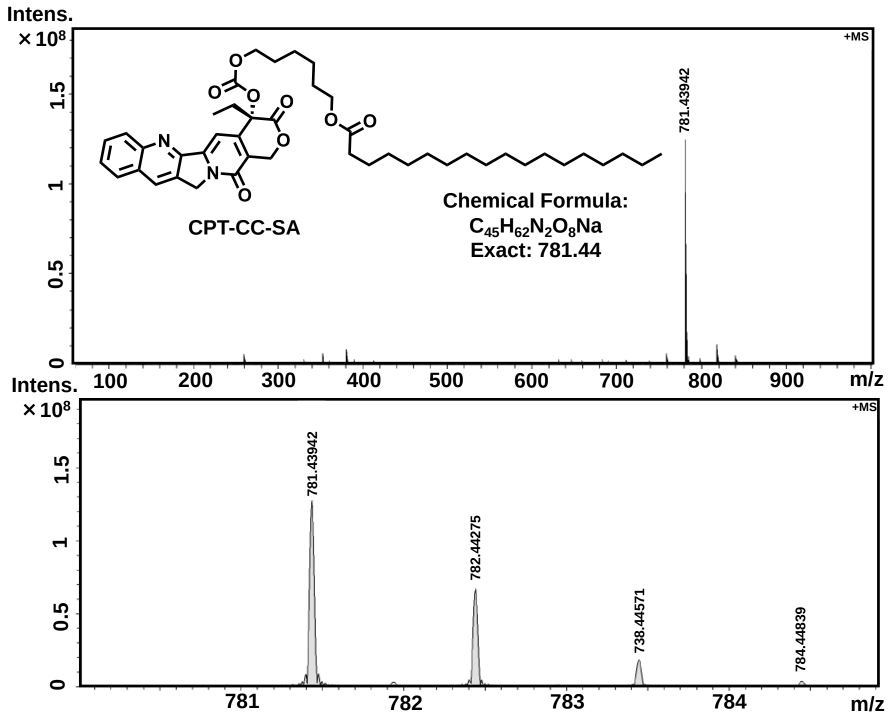

Figure S7. ESI-MS spectrum of CPT-CC-SA.

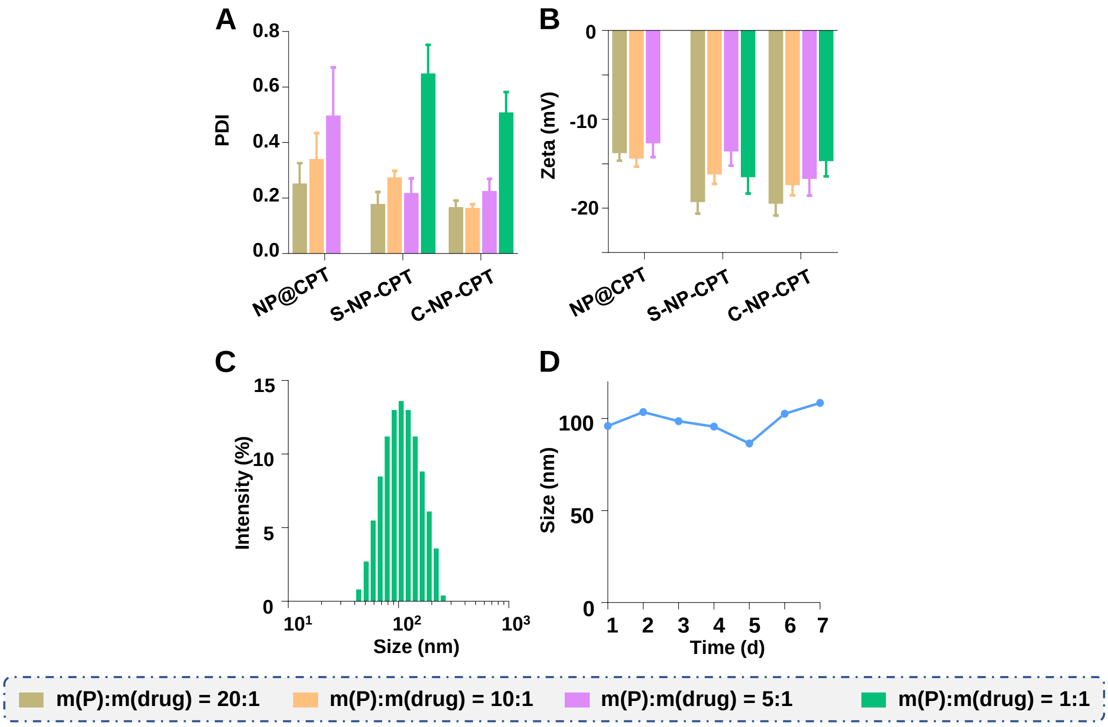

Figure S8. Characterization of S-NP-CPT. A, B, The PDI and Zeta-potential of S-NP-CPT by adjusting the quality comparison between CPT-SS-SA and mPEG$_{2000}$-DSPE. C, The average size of S-NP-CPT. D, Stability of S-NP-CPT.

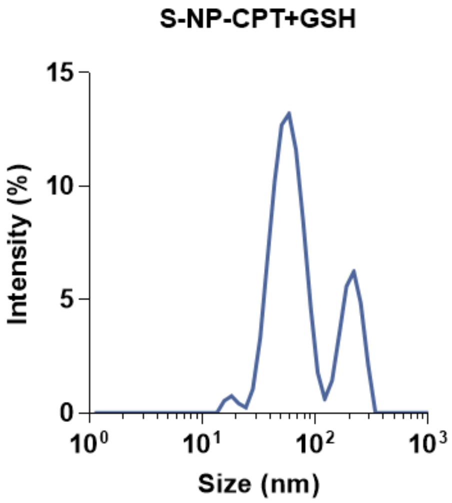

Figure S9. Representative particle size distribution of S-NP-CPT after GSH treatment.

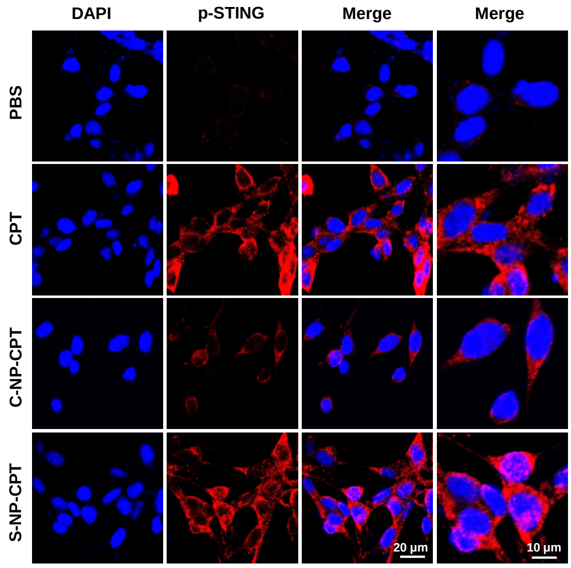

Figure S10. CLSM images of p-STING upon various treatments. Cell nuclei were stained with DAPI (blue), and p-STING was stained with Alexa Fluor 555 (red).

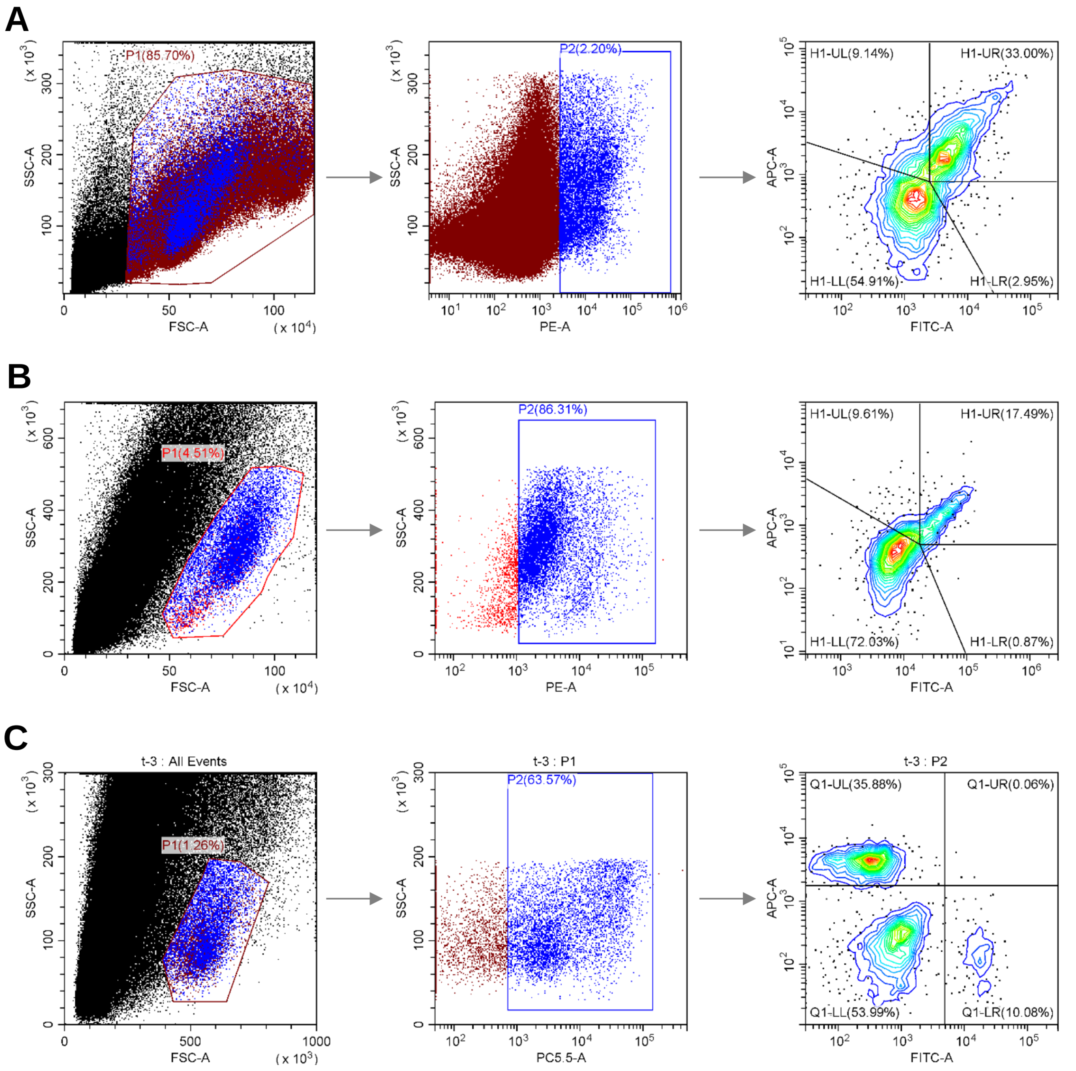

Figure S11. Gating strategy for flow cytometry analysis. A) After 3 days of treatment, the tumor draining lymph nodes (TDLNs) were digested and the dissociated cells were collected for flow cytometry analysis. Cells of interest were chosen on the basis of the size in the FSC/SSC plots. The mature DCs were designated as $CD11c^{+}CD80^{+}CD86^{+}$cells. B, C) After 3 days of treatment, tumor tissues were digested and dissociated cells were collected for flow cytometry analysis. Cells of interest were chosen based on the size in the FSC/SSC plots, and then the total number of T cells was determined by $CD3^{+}$ cell counts. $CD4^{+}$ and $CD8^{+}$ T cells were then gated within $CD3^{+}$ T cells (C). Dendritic cells (DC) were determined by $CD11c^{+}$ cell counts (B), mature DCs were designated as $CD11c^{+}CD80^{+}CD86^{+}$cells.

References

[1] R. Qi, Y. Wang, P. M. Bruno, H. Xiao, Y. Yu, T. Li, S. Lauffer, W. Wei, Q. Chen, X. Kang, H. Song, X. Yang, X. Huang, A. Detappe, U. Matulonis, D. Pepin, M. T. Hemann, M. J. Birrer, P. P. Ghoroghchian, *Nat Commun* **2017**, 8, 2166.

[2] a) F. Wang, H. Su, D. Xu, W. Dai, W. Zhang, Z. Wang, C. F. Anderson, M. Zheng, R. Oh, F. Wan, H. Cui, *Nat Biomed Eng* **2020**, 4, 1090-1101; b) L. Zhang, K. Shang, X. Li, M. Shen, S. Lu, D. Tang, H. Han, Y. Yu, *Adv Funct Mater* **2022**, 32, 2204589.
